# Can Pay-for-Performance Improve Quality and Reduce Health Disparities?

**DOI:** 10.1371/journal.pmed.0040216

**Published:** 2007-06-12

**Authors:** Katie Coleman, Richard Hamblin

## Abstract

The authors discuss a new study that examined whether pay-for-performance improves or worsens existing disparities in health between ethnic groups.

Pay-for-performance programs have been embraced by United States and United Kingdom policy makers and payors (those who pay for health-care services) as a means to improve the quality of health care. In fact, since the Institute of Medicine's 2001 report *Crossing the Quality Chasm* suggested realigning incentives to improve care [1], the UK's National Health Service (NHS) introduced pay-for-performance (P4P) contracts for all family practitioners. In the US, more than half of commercial health maintenance organizations have started using such contracts, and recent legislation requires that the Centers for Medicare and Medicaid Services do the same for Medicare [2].

Despite growing enthusiasm for P4P programs in the policy and commercial sectors, the evidence to support their effectiveness is weak. Only a handful of studies have directly examined the impact of financial incentives on improving health-care processes and outcomes, and the results of those studies are mixed (K. Coleman, K. L. Reiter, D. Fulwiler, unpublished data). Generally, studies show that modest improvements can be achieved on the measures that are explicitly incentivized, at least over the short term [3]. However, it is unclear whether the improvements are a result of the financial incentives themselves rather than simply the increased focus on services resulting from measurement of performance and publication of data [4].

In addition to the lack of evidence supporting P4P programs, there are also concerns about the possible unintended consequences of implementing P4P. For example, in their evaluation of the NHS P4P program, Doran and colleagues found that the strongest predictor of high achievement (i.e., where a family practice successfully met a number of pre-specified quality targets) was exception reporting—a practice where physicians can exempt certain patients from being included in their performance data due to medical or other reasons [5]. Exception reporting rates for physician practices ranged from 0% to 85.8%, with a median of 6%. In addition to creating incentives for dishonest reporting of data, or gaming, we are starting to see evidence that P4P may undermine other important quality initiatives such as reducing health disparities. In the NHS program, family practices with a high proportion of patients who were living in single-parent or low-income households were less likely to meet the quality targets [5].

**Linked Research Article**

This Perspective discusses the following new study published in *PLoS Medicine*:

Millett C, Gray J, Saxena S, Netuveli G, Khunti K, et al. (2007) Ethnic disparities in diabetes management and pay-for-performance in the UK: The Wandsworth Prospective Diabetes Study. PLoS Med 4(6): e191. doi:10.1371/journal.pmed.0040191

Based on a population-based longitudinal survey, Christopher Millett and colleagues concluded that pay-for-performance incentives for UK general practitioners had not addressed disparities in the management and control of diabetes between ethnic groups.

## A New Study on P4P and Health Disparities

In a new study published in *PLoS Medicine*, Christopher Millett and colleagues more fully explore the link between health disparities and P4P [6]. The authors provide the first look at the differential impact of the NHS P4P program on patients with diabetes from different ethnic groups, including black Caribbean, black African, Indian, Pakistani, Bangladeshi, white Irish, and white British.

Using disease registers in Wandsworth, an ethnically diverse borough of London, the authors studied performance data on treatment targets for diabetes management, examining the management and control of glycated hemoglobin, cholesterol, and blood pressure before and after the implementation of the NHS P4P program. Though improvements in process and outcomes were seen across most ethnic groups, the black Caribbean group had substantially lower improvements in glycated hemoglobin levels and blood pressure control than the white British group, reinforcing pre-existing disparities.

The authors also found some evidence that different ethnic groups received different treatment for their hyperglycemia: black and South Asian patients more often received oral hypoglycemic agents and white patients more often received insulin prescriptions.

Limitations in the study design—including the inability to adjust for clinically relevant confounders such as disease severity, duration, and complications—make it difficult to interpret these findings. The differential prescribing patterns and outcomes could be attributable to anything from medical necessity or patient preference to systematic differences in self-management behaviors or provider bias. Despite these caveats, the study does prompt continued exploration of the intersection of popular P4P programs and their impact on health disparities. The study also encourages us to think more deeply about how incentive programs may unintentionally amplify existing differences in health between different ethnic groups.

## Policy Implications

As major P4P projects such as Medicaid programs and others begin [7], we have the opportunity to integrate important lessons learned. Early evidence does not support the position of some commentators who believed P4P could be a “silver bullet” to improve health-care quality and reduce costs [8]. Instead, as Millett and colleagues show, P4P alone may not be able to drive equitable improvement in health-care quality.

How can we ensure that P4P programs help us to achieve the quality goals that we have set for ourselves, including a reduction in health disparities? First, we must ensure that performance-based pay is not the sole mechanism driving quality improvement. Instead, if we contextualize P4P as part of a larger quality improvement effort, then we can leverage demonstrated quality improvement paradigms, such as the Chronic Care Model [9], and use P4P to reinforce program goals.

Next, the literature shows us that we must be clear about what processes and outcomes are expected to change with P4P, and we must align financial incentives and improvement efforts with those expectations [3]. If we want to reduce health disparities, we have to focus efforts on progress toward that goal. Millett and colleagues show us that we cannot assume that a quality improvement effort will benefit all patients equally.

Finally, one way to reduce such disparities is to create opportunities for local goal setting. Though large scale policy-making bodies such as Medicare and the NHS set broad improvement agendas, the unique characteristics of regions such as Wandsworth highlight the importance of flexible performance measures. Health care is, after all, local. We need to recognize the diversity of contexts within which health services operate to achieve the improvements in quality and the reduction in disparities to which we aspire.

## Abbreviations

NHS: National Health Service
P4P: pay-for-performance

## References

[pmed-0040216-b001] Institute of Medicine (2001). Crossing the quality chasm: A new health system for the 21st century.

[pmed-0040216-b002] Rosenthal MB, Landon BE, Normand SL, Frank RG, Epstein AM (2006). Pay for performance in commercial HMOs. N Engl J Med.

[pmed-0040216-b003] Casalino LP, Elster A (2007). Will pay-for-performance and quality reporting affect health care disparities?. Health Aff (Millwood).

[pmed-0040216-b004] Hamblin R (2007). Publishing “quality” measures: How it works and when it doesn't. Int J Qual Health Care.

[pmed-0040216-b005] Doran T, Fullwood C, Gravelle H, Reeves D, Kontopantelis E (2006). Pay-for-performance programs in family practices in the United Kingdom. N Engl J Med.

[pmed-0040216-b006] Millett C, Gray J, Saxena S, Netuveli G, Khunti K (2007). Ethnic disparities in diabetes management and pay-for-performance in the UK: The Wandsworth Prospective Diabetes Study. PLoS Med.

[pmed-0040216-b007] Llano K, Rothstein J, Dyer MB, Bailit M, for the Center for Health Care Strategies (2007). Physician pay-for-performance in Medicaid: A guide for states. http://www.chcs.org/publications3960/publications_show.htm?doc_id=471272.

[pmed-0040216-b008] Rosenthal MB, Dudley RA (2007). Pay-for-performance: Will the latest payment trend improve care?. JAMA.

[pmed-0040216-b009] Wagner EH, Austin BT, Davis C, Hindmarsh M, Schaefer J (2001). Improving chronic illness care: Translating evidence into action. Health Aff (Millwood).

